# Lack of evidence of *WNT3A *as a candidate gene for congenital vertebral malformations

**DOI:** 10.1186/1748-7161-2-13

**Published:** 2007-09-23

**Authors:** Nader Ghebranious, Cathleen L Raggio, Robert D Blank, Elizabeth McPherson, James K Burmester, Lynn Ivacic, Kristen Rasmussen, Jennifer Kislow, Ingrid Glurich, F Stig Jacobsen, Thomas Faciszewski, Richard M Pauli, Oheneba Boachie-Adjei, Philip F Giampietro

**Affiliations:** 1 Molecular Diagnostic Research Laboratory, Marshfield Clinic, Marshfield, Wisconsin, USA; 2 Department of Pediatric Orthopedics, Hospital for Special Surgery, New York, New York, USA; 3 University of Wisconsin Medical School, Madison, Wisconsin, USA and Geriatrics Research, Education, and Clinical Center, William S. Middleton Veterans Administration Medical Center, Madison, Wisconsin, USA; 4 Department of Medical Genetic Services, Marshfield Clinic, Marshfield, Wisconsin, USA; 5 Center for Human Genetics, Marshfield Clinic Research Foundation, Marshfield, Wisconsin, USA; 6 Office of Scientific Writing and Publications, Marshfield Clinic Research Foundation, Marshfield, Wisconsin, USA; 7 Department of Orthopedic Spine Surgery, Marshfield Clinic, Marshfield, Wisconsin, USA; 8 University of Wisconsin-Madison, Clinical Genetic Center, Madison, Wisconsin, USA; 9 Adult and Pediatric Spine Surgery, Hospital for Special Surgery, New York, New York, USA

## Abstract

**Background:**

Prior investigations have not identified a major locus for vertebral malformations, providing evidence that there is genetic heterogeneity for this condition. *WNT3A *has recently been identified as a negative regulator of Notch signaling and somitogenesis. Mice with mutations in *Wnt3a *develop caudal vertebral malformations. Because congenital vertebral malformations represent a sporadic occurrence, linkage approaches to identify genes associated with human vertebral development are not feasible. We hypothesized that *WNT3A *mutations might account for a subset of congenital vertebral malformations.

**Methods:**

A pilot study was performed using a cohort of patients with congenital vertebral malformations spanning the entire vertebral column was characterized. DNA sequence analysis of the *WNT3A *gene in these 50 patients with congenital vertebral malformations was performed.

**Results:**

A female patient of African ancestry with congenital scoliosis and a T12-L1 hemivertebrae was found to be heterozygous for a missense variant resulting in the substitution of alanine by threonine at codon 134 in highly conserved exon 3 of the *WNT3A *gene. This variant was found at a very low prevalence (0.35%) in a control population of 443 anonymized subjects and 1.1% in an African population.

**Conclusion:**

These data suggest that *WNT3A *does not contribute towards the development of congenital vertebral malformations. Factors such as phenotypic and genetic heterogeneity may underlie our inability to detect mutations in *WNT3A *in our patient sample.

## Background

Congenital vertebral malformations are phenotypically and etiologically heterogeneous. Their estimated incidence is between 0.5 to 1/1000 [1-4]. Vertebral malformations may represent an isolated finding, occur in association with other renal, cardiac, or spinal cord malformations, or occur as part of an underlying chromosome abnormality or syndrome. These include, but are not limited to, hemifacial microsomia, Alagille, Jarcho-Levin, Klippel-Feil, Goldenhar,, basal cell nevus, trisomy 18, diabetic embryopathy and VACTERL (vertebral, cardiac, renal, limb anomalies, anal atresia, tracheo-esophageal fistula) syndromes. Vertebral malformations most commonly include hemivertebrae, vertebral bars, supernumerary vertebrae, butterfly vertebrae, and wedge-shaped vertebrae.

Congenital scoliosis is caused by segmentation defects such as fused vertebrae, vertebral body formation defects, and mixed defects in which both types of lesions are encountered [3,4]. Each of these conditions may cause development of a spinal curve based on asymmetric growth. The severity of the curve is related to the type of defect and whether or not the primary problem is accompanied by any compensatory developmental changes.

Vertebral segmentation proceeds through a clock and wave front mechanism under Notch signaling control. Negative feedback through the canonical Wnt/β catenin pathway via Axin 2 modulates the process [5,6]. Based on mouse-human synteny analysis, a series of candidate genes known to cause vertebral malformations in the mouse have been identified [5-8]. One gene, *Wnt3a*, is necessary for generation of the posterior portion of the neuraxis, as knockout mice fail to develop a tailbud and are truncated from a point slightly anterior to the hindlimbs [9]. This gene is a member of a moderate-sized multigene family comprised of at least 12 members in humans and the mouse. Genes in this family function both in establishing the body plan in development and as potential oncogenes [10]. *Wnt3a *has been proposed to be a major controlling gene in the oscillation of Notch signaling that is necessary for segmentation to occur [5].

Since isolated congenital vertebral malformation most often represents a sporadic occurrence within a particular family, it is virtually impossible to utilize traditional linkage approaches to identify causative genes. This makes candidate gene analyses a viable alternative method to study this condition. We hypothesized that mutations in *WNT3A *may be associated with the development of isolated vertebral malformations and congenital scoliosis. In order to test this hypothesis we undertook a pilot study and performed DNA sequence analysis in a heterogeneous cohort of 50 patients with congenital vertebral malformations.

## Methods

### Vertebral malformation subjects

Fifty children and adults with radiographic evidence for congenital segmental vertebral malformations were identified from the orthopedics and clinical genetic practices at Marshfield Clinic, Marshfield, Wisconsin, the Pediatric Orthopedics Department at the Hospital for Special Surgery, New York, New York, and the Bone Dysplasia Clinic at the University of Wisconsin, Madison.

Medical records of each patient were reviewed to determine if cardiac, renal, or spinal cord malformations were previously identified. Blood samples or buccal swabs were obtained from index cases (probands) and parents (when available) for subsequent DNA analysis. Five tissue samples were obtained from stillborn infants through the Wisconsin Stillbirth Program. Subjects' vertebral malformations were classified as previously described [11].

### Mutational Analysis

Genomic DNA was amplified and the products were purified using the QIAquick PCR purification kit (QIAGEN, Inc., Valencia, California) and sequenced using either unidirectional or bi-directional di-deoxy sequencing with the DYEnamic ET Terminator Cycle Sequencing Kit (Amersham Biosciences Corp., Piscataway, New Jersey) on the PRISM 3100 Genetic Analyzer (Applied Biosystems, Foster City, California). Sequence analysis was performed using DNASTAR software (DNASTAR, Madison, Wisconsin). The same primers were used for amplification and sequencing. Primers and product sizes are shown in Table 1.

**Table 1:** Primers and corresponding product sizes

**Primer**	**Sequence**	**Length**	**Product size (bp)**
WT-P-E1(F2)	CAGCACGTCCTCAGACACAC	20	581
WT-P-E1(R2)	CCGTCAAGGAAAACCAACC	19	
WTE2(F)	TAGCCTGCTCATCTGTGTCG	20	367
WTE2(R)	GCTAGGCGGGAGACTCTGT	19	
WTE3(F)	AATGGGCTAAGACCCCTGAC	20	390
WTE3(R)	GAGGCTCCTTTTTCCCAAG	19	
WTE4(F)	CAGGCGACATGTAATGCTGT	20	644
WTE4(R)	TTTAGGTGGGAGTCCTGCTC	20	
• WNTE3Mut(F)	**ACGTTGGATG**ACACGCTCATGTGCAGAAGG	30	94
• WNTE3Mut(R)	**ACGTTGGATG**TACAGCCACCCCACTTCCAG	30	
• WNTE3Mut(UE)	TGTGCAGAAGGCACGGCC	18	
• WNT-pr(F)	**ACGTTGGATG**TATCCGAGTGGGGCCATCGC	30	98
• WNT-pr(R)	**ACGTTGGATG**CGCCAGCTCCCAGGGCCCG	29	
• WNT-pr(UE)	CCGAGAGCGTGAGCGCC	17	

The amplification and extension primers used in the homogeneous mass extend reaction are bulleted. The 10-mer tag used that increases the mass of the amplification primers so that they would fall outside the mass spectrophotometer range of analysis are bolded. UE denotes the unextended primers used in the mass extend reaction.

DNA sequence variants identified in subjects with vertebral malformations were compared to the UCSC goldenPath May 2004 sequence assembly [12].

Allele frequencies were determined in the control population using the homogeneous mass extend reaction and the matrix-assisted laser desorption/ionization time-of-flight mass spectrophotometer (Sequenom) [13]. A panel of 443 subjects (360 of whom were from the CEPH diversity panel) was tested for the *WNT3A *variant. The panel consisted of 126 subjects from African ancestry, 299 were Caucasians, 12 were Oceanic, and 6 were Asians. A panel of 23 anonymized DNA samples was used to determine the frequency of the *WNT3A *C/G promoter polymorphism, respectively.

## Results

**Figure 1:**
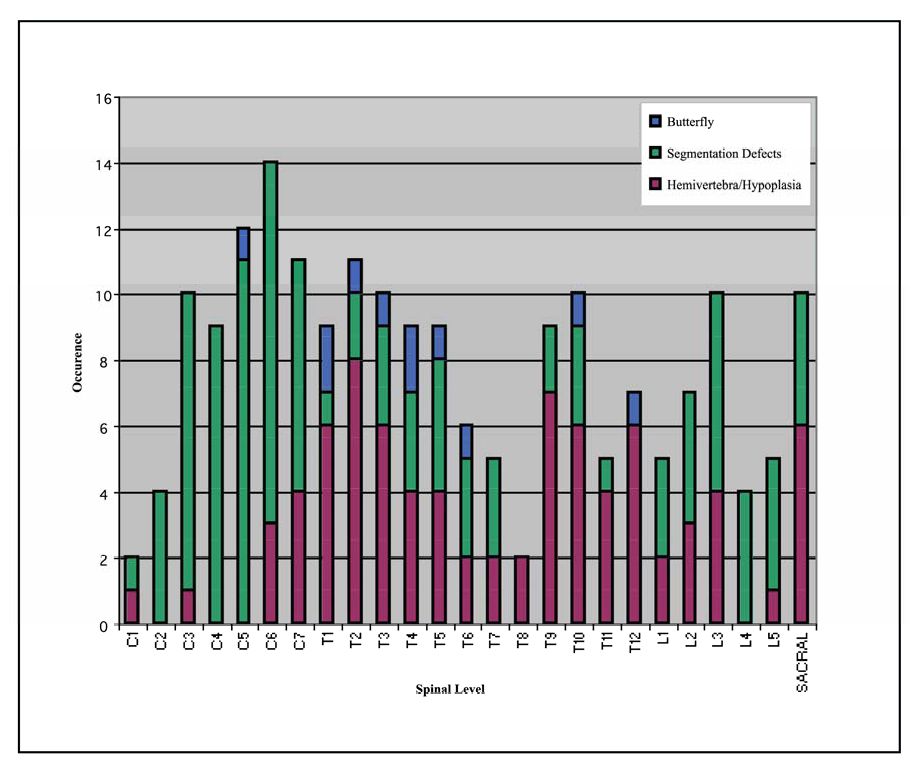
Relative occurrence of vertebral malformations by spinal level and type.

Vertebral malformations were classified as butterfly vertebra, segmentation defect, or hemivertebra/hypoplasia. The vertebral malformations spanned the length of the entire spine and were classified as butterfly vertebrae, segmentation defect, hypoplasia and hemivertebrae [4]. Butterfly vertebrae were defined by the presence of a sagittal cleft. Segmentation defects include defects arising from intervertebral discs such as block vertebrae and unsegmented vertebral bars. The vertebral malformations spanned the length of the entire spine and are illustrated (figure 1). The malformation characteristics of the study sample are highlighted in Table 2.

**Table 2:** Malformation characteristics of study sample (n = 50)

**Category of diagnosis of vertebral malformation**	**Number**
Isolated vertebral malformation(s)	15
Multiple congenital anomalies	11
VACTERL syndrome	9
Klippel-Feil syndrome	8
Facio-auriculo-vertebral spectrum and Goldenhar	3
Jugulolymphatic obstruction sequence	1
Sacral agenesis	1
Spondylothoracic dysplasia	1
Spondylocostal dysostosis	1

**Figure 2:**
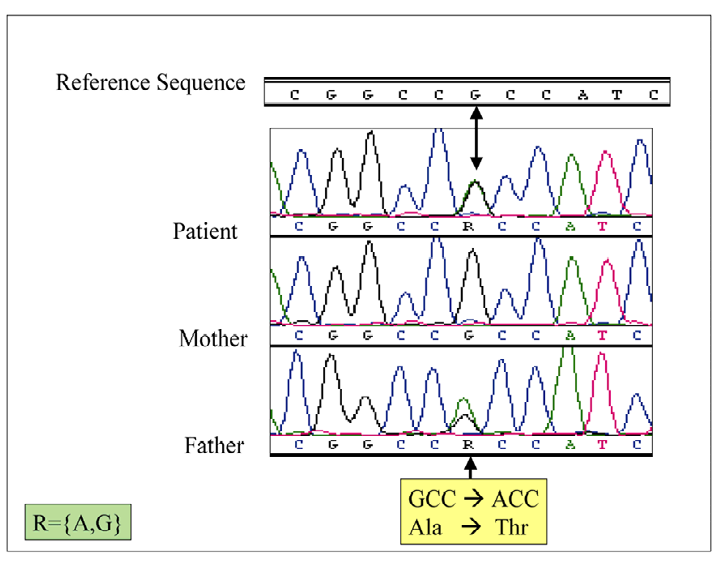
Sequencing chromatogram of patient with T12-L1 hemivertebrae illustrating *WNT3A *exon 3 polymorphism.

To analyze the *WNT3A *gene in the selected vertebral malformation cohort, a total of four segments in the *WNT3A *gene were amplified and sequenced in each patient. The sequencing covered the four exonic regions, splice junctions, and 381 bp of the promoter region. A 23-month-old female child of Cape Verde African ancestry with an isolated T12-L1 hemivertebrae and congenital scoliosis with a 38° lumbar scoliotic curve was found to be heterozygous for a **G**CC to **A**CC missense variant at codon 134 (UCSC position chr1:224,545,178 May 2004 assembly) of the *WNT3A *gene (figure 2), resulting in the substitution of alanine by threonine. The patient's clinically asymptomatic father was also heterozygous for the missense variant, whereas her mother was not. This exon is highly conserved, whereas codon 134 is less conserved in this exon. Interestingly, the alanine amino acid at codon 134 is conserved in mouse, rat, dog and opossum, whereas in elephant, chicken, *Xenopus tropicalis *and tetradon, alanine is replaced by threonine. To check for the prevalence of this variant in reference population, a total of 890 chromosomes from 445 subjects were tested for the *WNT3A *mutation, of which 133 were of African origin, 299 were Caucasians, and 11 were Asians. Only three subjects of African origin (Yoruba, Mbuti, and Bantu N.E.) were heterozygous for the variant. The overall prevalence of the variant in the tested reference population is 0.35%.

Moreover, a novel C/G single nucleotide variant was also found in the promoter region of the *WNT3A *gene (UCSC position chr1:224,501,511 May 2004 assembly). The patient cohort had an average heterozygosity of 0.130 and allele frequencies of 0.93 and 0.07 for the C and G alleles, respectively. In order to determine the significance of this variant, we typed a panel of 23 anonymized reference DNA samples of Caucasian ancestry. The average heterozygosity for this variant in the reference cohort was calculated to be 0.159 and the allele frequencies were 0.91 and 0.09 for the C and G alleles, respectively. The genotype distribution for this variant did not differ significantly between the patient's and reference cohorts (Chi-squared = 0.001, p-value = 0.97) and were in Hardy-Weinberg equilibrium in both cohorts. We conclude that this variant likely represents a polymorphism.

## Discussion

In a series of 50 patients with a broad array of vertebral malformations encompassing the entire length of the spinal column, we only identified a non-synonymous variant in exon 3 of *WNT3A*, resulting in substitution of alanine for threonine. Our observation of this variant with an overall frequency of 0.35% in the control population studied, and a frequency of 1.1% of African chromosomes included in the control population suggest and the observation that the patient's clinically asymptomatic father harbors the same variant strongly suggest that this variant may represent a polymorphism in the African population. Based on the data obtained from our pilot study, mutations in the *WNT3A *gene do not appear to be a common etiologic factor in the development of congenital vertebral malformations.

Patient heterogeneity for vertebral malformations and heterogeneity of candidate genes represent major limitations in genetic analyses. It is possible that mutations in *WNT3A *would have been identified if we had sequenced a cohort of patients characterized by having caudal vertebral malformation phenotypes. Because congenital vertebral malformations represent a rare condition, collection of a phenotypically homogeneous sample is not practical and would require a greater amount of time. DNA sequence analysis was limited to the *WNT3A *promotor, exons, and intron-exon boundaries. Sequence variants in the introns that could potentially contribute to abnormal transcription would not have been detected. Copy number variations in *WNT3A *will not have been detected by this methodology. Other genes that regulate the expression of *WNT3A *have not been examined.

Evidence for genetically heterogeneous etiologies of vertebral malformations includes the identification of mutations in *DLL3 *in patients with spondylocostal dysostosis [14,15] and mutations identified in *JAG 1 *in patients with Alagille syndrome [16]. Recently, a few DNA coding sequence alterations with uncertain clinical significance in *PAX1 *and *DLL3 *have been identified in a cohort of patients with phenotypically characterized vertebral malformations [17-20]. Our inability to detect mutations in *WNT3A*, as well as other candidate genes studied, may reflect differences in the regulation of somitogenesis in humans as compared to mice. It is possible that mutations in *WNT3A *are lethal in humans. Although our pilot study included 5 stillbirth samples and no mutations in *WNT3A *were detected, a larger cohort of stillbirth samples with vertebral malformations would be needed to determine whether mutations in *WNT3A *are prevalent among stillbirths with congenital vertebral malformations. Tissue mosaicism for a specific mutation and/or epigenetic factors may have an influence in the development of vertebral malformations in humans. Vertebral malformations may have greater environmental components than genetic components to their origin. A relationship between abnormal distribution of the intersegmental arteries and vertebral malformations was observed in 4 of 11 human embryos and fetuses with congenital vertebral malformations, suggesting the possibility that abnormalities in vascularization during the resegmentation process may contribute towards the development of congenital vertebral malformations [21].

Clinical relevance in identifying mutations associated with the development of vertebral malformations and congenital scoliosis is to develop a molecular classification system that could provide prognostic information regarding the severity of progression of congenital scoliosis and the presence of associated birth defects to practitioners and families. Although congenital scoliosis and idiopathic scoliosis represent dichotomous processes, namely a failure to develop normal vertebral structures for the former and a failure to lose normal vertebral structures for the latter, there is evidence that similar genetic factors may play a role in both conditions [22]. Identification of gene mutations which are associated with the development of congenital scoliosis may also help improve our understanding of the pathogenesis of idiopathic scoliosis.

In summary, we have been unable to detect mutations in the *WNT3A *gene in a pilot study of 50 patients with vertebral malformations encompassing the entire spine. This study illustrates the problems posed by attempting to design studies aimed at identification of patterning genes contributing towards the development of vertebral malformations. Future studies aimed at investigating the etiology of congenital vertebral malformations should be aimed at the study of additional candidate genes in a more homogeneous patient population.

## Competing interests

The author(s) declare that they have no competing interests.

## Authors' contributions

PFG, NG, CLR, RDB, EM, JKB, IG, FSJ, TF and OBA designed or assisted in the study design. NG, LI, and JK performed laboratory analysis of DNA samples obtained from patients. NG, CLR, RDB, EM, JKB, LI, KR, and JK assisted in the interpretation of data. PFG recruited patients. PFG, CLR, RDB, EM, KR, FSJ, TF, RMP, and OBA assisted in the analysis of phenotypic data. RDB assisted in the analysis of molecular data and PFG, IG, RMP performed the analysis of data. All authors assisted in the preparation and/or review of the manuscript and have read and approved the final manuscript.
